# Anterior cruciate ligament reconstruction is associated with increased corticospinal excitability and rate of force development

**DOI:** 10.1186/s13102-026-01577-0

**Published:** 2026-02-09

**Authors:** Stefano Scarano, Antonio Caronni, Alessandra Menon, Viviana Rota, Maurizio Amadei, Laura Perucca, Elena Brevi, Alessio Maione, Paolo Ferrua, Luigi Tesio, Pietro Simone Randelli

**Affiliations:** 1 Department of Neurorehabilitation Sciences, IRCCS Istituto Auxologico Italiano, Ospedale San Luca, Milan, Italy; 2 Department of Biomedical Sciences for Health, University of Milan, Milan, Italy; 3 Department of Biomedical Sciences for Health, Laboratory of Applied Biomechanics, University of Milan, Milan, Italy; 4 Department of Clinical and Community Sciences, Graduate School of Health Statistics and Biometrics, University of Milan, Milan, Italy; 5 U.O.C. 1° Clinica Ortopedica, ASST Gaetano Pini-CTO, Milan, Italy; 6 REsearch Center for Adult and Pediatric Rheumatic Diseases (RECAP-RD), Department of Biomedical Sciences for Health, Università degli Studi di Milano, Via Mangiagalli 31, Milan, 20133 Italy

**Keywords:** ACL reconstruction, Transcranial magnetic stimulation, Non-invasive brain stimulation, Joint torque, Quadriceps hypotrophy

## Abstract

**Background:**

After anterior cruciate ligament reconstruction, asymmetries in central activation are suspected to prevent complete functional recovery. This cross-sectional study investigated the motor function of both lower limbs in ACLR patients using morphological, mechanical, and neurophysiological measures after surgical repair with a semitendinosus-gracilis graft.

**Methods:**

Ten male patients (age 20–31 years; 6/4 right/left knee surgery; 6–12 months after ACLR) were recruited. Muscle trophism was quantified through ultrasound estimates of quadriceps volume and mid-thigh circumference; knee extensors’ rate of force development (RFD), maximum torque and voluntary activation (interpolated twitch technique, ITT) were assessed through dynamometry during maximal isometric effort; spinal excitability was measured with the Soleus H-reflex; transcranial magnetic stimulation was used to assess corticospinal excitability (resting motor threshold (rMT) and recruitment curve of motor evoked potentials (MEP) during submaximal contraction) and intracortical excitability (short-interval intracortical inhibition (SICI)) of the Vastus medialis (VM) and Tibialis anterior (TA) muscles.

**Results:**

The quadriceps muscle on the operated side showed significant volume loss (mean [SD] of 2264.6 [345.1] cm³ and 2082.9 [386.2] cm³ for the non-operated and operated sides, respectively; *p* = 0.035) and mid-thigh circumference (52.2 [2.7] cm and 50.2 [3.3] cm; *p* = 0.035). In the VM recruitment curves, the increase in MEP amplitude with increasing stimulation intensity was steeper on the operated side (*p* = 0.001). The operated limb also showed a higher RFD (*p* = 0.026). No inter-limb differences were found for the remaining outcomes.

**Conclusions:**

The steeper rise of knee extensor torque, paralleled by an increased corticospinal excitability of the operated side VM muscle, suggests that an increased drive from the motor cortex is needed to engage the quadriceps in fast contractions following ACLR. This may represent a compensatory phenomenon aimed at counteracting the decline in muscle power associated with reduced muscle mass and altered quadriceps morphology.

**Trial registration:**

ClinicalTrials.gov NCT04837417 (submitted On 2021-03-31).

**Supplementary Information:**

The online version contains supplementary material available at 10.1186/s13102-026-01577-0.

## Background

Anterior cruciate ligament (ACL) tears are among the most common knee injuries, particularly in young active individuals and athletes [1]. The ACL is the main stabiliser of the knee joint, with its deficiency resulting in anterior and rotatory instability [2]. ACL reconstruction (ACLR) is a primary treatment option [3], especially for patients engaged in sports [4]. ACLR is mainly performed using autografts (hamstrings tendons or bone-patella tendon-bone autografts) [4] and is primarily aimed at restoring knee stability [5], allowing patients to return to complete, unrestricted motor activities [6]. Decreased occurrence of secondary injuries to the joint and lowered incidence of progressive post-traumatic osteoarthritis are still debated outcomes [7, 8]. After ACLR, patients may still report suboptimal physical function despite appropriate pre- and postoperative rehabilitation and restoration of knee stability. More specifically, a relevant percentage of patients with ACLR report kinesiophobia (i.e., an excessive, irrational, and debilitating fear of physical movement and activity), a lack of self-efficacy and confidence in the operated knee, and fear of reinjury [9, 10]. Indeed, outcome measures have been developed to specifically assess psychological readiness to return to sport following ACLR, like the Anterior Cruciate Ligament-Return to Sport After Injury (ACL-RSI) scale [11]. These subjective outcomes are paralleled by evidence of persistent quadriceps atrophy [12], long-lasting deficits in quadriceps strength and neural activation [13, 14], and modifications in gait kinematics and dynamics [15]. Complete recovery of athletic skills is not always achieved. Incomplete recovery after ACLR may be responsible for the increased risk of reinjury (either a second injury to the ipsilateral graft or a new injury to the contralateral ACL, with a 15% overall reinjury rate in ACL-reconstructed athletes) [16] and the low proportion of athletes who successfully return to sports after surgery [17].

Previous research suggests that persistent deficits after ACLR are driven by central nervous system (CNS) modifications. This neuroplasticity is complex: while often maladaptive (contributing to muscle force inhibition) [18], it may also be compensatory. These neurophysiological adaptations stem from multiple factors, including deafferentation (loss of ACL mechanoreceptors [19]), joint immobilisation, pain, and the motor learning processes induced by rehabilitation [18]. There is evidence supporting the existence of differences in the excitability of the cortico-spinal pathways between individuals with ACLR and uninjured controls [20]. The literature has documented reduced quadriceps strength associated with both muscle atrophy and decreased voluntary activation (VA) in subjects with reconstructed and non-reconstructed ligaments compared to healthy controls [14, 21]. The ability to generate muscle force rapidly, measured as the rate of force development (RFD), has shown persistent inter-limb asymmetries after ACLR, with the highest inter-limb asymmetry in knee extensor RFD occurring in the 4–7 months after surgery [22]. Studies investigating the excitability of the corticospinal tract using transcranial magnetic stimulation (TMS), although yielding conflicting findings [21], have reported a bilateral increase of the quadriceps motor threshold when compared to healthy controls, and slightly reduced amplitudes of the quadriceps motor evoked potentials (MEPs) on the injured side compared to the uninjured one [23]. Intracortical excitability seems to be affected, too, since a reduced short-interval intracortical inhibition (SICI) has been observed on the hemisphere contralateral to the injured knee [23]. Regarding spinal reflexes, a meta-analysis found no difference in the H-reflex of the Vastus medialis (VM) between injured and healthy limbs [21].

These findings suggest that, following ACL injury and subsequent ACLR, motor pathway adaptations are located along the corticospinal tract, likely originating within the primary motor cortex. Research in this field still needs refinement. No studies assessed intracortical, corticospinal, and spinal reflex excitability, muscle VA, muscle strength, RFD, and muscle volume in the same individuals. It is also worth noting that most previous studies are characterised by highly heterogeneous patient samples. This is a critical confounder, as recent evidence suggests that corticospinal excitability evolves over time after ACLR [24, 25]. Yet most studies have aggregated patients with markedly different recovery times, as evidenced by large standard deviations: e.g., 48.10 ± 36.17 months [26], 44.47 ± 36.58 months [27], 33.9 ± 26.1 months [28]. Likewise, previous studies did not distinguish between surgical techniques, with samples comprising patients with either graft type in varying proportions [25–33]. Furthermore, many studies did not control for injuries to joints other than the knee (e.g., chronic ankle instability [34]) and previous muscle strain injuries (e.g., hamstring strain injury [35]). The above flaws were addressed in the present study.

In addition, the hypothesis was tested that in ACLR patients, CNS plasticity also affects leg muscles (e.g. Tibialis anterior (TA) and Soleus), not only thigh muscles (e.g., VM), given that (a) the mechanical coupling between limb segments implies that kinematic or kinetic alterations at the ankle or hip inevitably propagate to the knee, and vice versa, and (b) motor control relies on synergies and even ostensibly mono-articular movements (such as knee extension) require the coordination of multiple body segments [36]. Therefore, it is reasonable to hypothesise that following ACL rupture and reconstruction, even leg muscles must adapt to the altered biomechanics of the knee.

## Methods

This multicentre cross-sectional observational study is part of a larger study aimed at assessing lower-limb function in ACLR patients using morphological, mechanical, and neurophysiological measures.

Given the physiopathological nature of this study, strict inclusion and exclusion criteria were applied to ensure a highly homogeneous sample. This minimisation of inter-subject variance intrinsically enhances statistical power; therefore, a sample size of 10 participants was considered sufficient even in the absence of a formal a priori power analysis. The rationale for the criteria is detailed in the following paragraphs.

*Inclusion criteria*:

- *Demographics*,* clinical history*,* anthropometric parameters*,* consensus*.

- Only male adults aged between 18 and 35 years were recruited. The decision to include only one sex was motivated by the known inter-sex differences in knee anatomy and biomechanics [37–40], which may also contribute to the observed difference in ACL injury incidence between men and women [41]. It is of importance here that inter-sex differences in corticospinal excitability and RFD after ACLR have recently been described [42], further supporting the decision to consider participants of only one sex in this exploratory study.
- Only patients who underwent arthroscopic anterior cruciate ligament reconstruction using a semitendinosus-gracilis autograft were eligible. While no gold standard for graft selection has yet been established [43–45], restricting recruitment to a single graft type was necessary to ensure sample homogeneity (possibly at the expense of generalizability to other types of surgery).
- Patients were recruited between 6 and 18 months after surgery. Similar criteria have been adopted in previous studies investigating ACLR recovery [29]. This time interval was based on evidence that most quadriceps strength deficits are still evident 6 months after ACLR [46] and that patients who fail to meet return-to-activity criteria at 6 months post-surgery are more likely to exhibit long-lasting deficits [47].
- Only patients who had recovered an active knee flexion ≥ 70° on the operated side were eligible. A flexion angle below this cutoff suggests that the surgical and rehabilitation treatment has been ineffective, possibly indicating the need for arthroscopic release [48].
- Patients had to have a pre-injury score of 5 or greater out of 10 on the Tegner Activity Scale [49].
- They had to have a normal weight, i.e., a body mass index (BMI) between 18.5 and 24.9 kg/m².
- They had to understand the study’s instructions and declare their willingness to participate and sign the informed consent form.

b) *Homogeneity of surgery*.
  Recruitment was restricted to patients operated on by a single surgical team at the Istituto Gaetano Pini, a teaching Orthopaedic Institute in Milan, using a standardised semitendinosus-gracilis autograft technique. The surgical procedure is described in section 1 of the Supplementary Materials. In section 2 of the Supplementary Materials, the recommendations patients received regarding the rehabilitation protocol to follow during outpatient physical therapy after ACLR are reported.

*Exclusion criteria*.

- *Orthopaedic and medical criteria*.

Patients were excluded if any other major trauma had occurred in the past, and if a major ligamental or meniscal lesion or reconstruction was reported in the surgical report or in the anamnesis. Therefore, the exclusion criteria were:

- Other previous major traumatisms or surgery to either lower limb.
- Any major surgery associated with anterior cruciate ligament reconstruction (e.g., osteotomy, reconstruction of other ligaments) or meniscal surgery involving the removal (meniscectomy) of more than 30% of meniscal volume or the meniscal roots.
- A relevant knee axial deviation, as defined by > 5° of varus or valgus.
- Any significant medical co-morbidity (e.g., infections with permanent clinical outcomes, rheumatic disorders, diabetes, osteoporosis, tumours, neurological disorders, vascular disorders, heart disorders).

b) *TMS incompatibility*.

Furthermore, patients were excluded from the study if they had absolute or relative contraindications to TMS:

- History of seizures or epilepsy in the patient or in a first-degree relative.
- Presence of intracranial metallic or magnetic pieces.
- Presence of pacemakers and other implantable medical devices (e.g., cochlear implants).
- Medications that may lead to a reduction in seizure threshold.
- History of substance abuse or alcohol abuse.

c) *Contraindication to muscle VA testing*.

- Patients on anticoagulant therapy were excluded, as it was considered a contraindication to muscle electrical stimulation.

### Screening and recruitment

All patients who underwent ACLR surgery between 2020 and 2023 were considered. According to the clinical records, patients who did not meet the inclusion and exclusion criteria were excluded from the study. The remaining patients were contacted, and their criteria were verified. Those who met the criteria were invited to participate in the study.

Following recruitment, the assessment commenced with a clinical evaluation conducted at the Orthopaedic Institute in Milan. The following instrumental assessment was conducted at the Neurorehabilitation Department of the IRCCS Istituto Auxologico Italiano in Milan. Instrumental assessments were conducted on two separate days: anthropometric measurements were followed by quadriceps strength and voluntary activation testing on one day, and neurophysiological testing was conducted on the other day.

### Clinical assessment

The clinical evaluation consisted of a comprehensive orthopaedic assessment performed by an orthopaedic surgeon experienced in ACLR surgery. The assessment aimed to verify the inclusion and exclusion criteria and to conduct a comprehensive examination of the operated knee joint (range of motion, pain during passive and active movement, and knee stability tests).

### Anthropometric measurements

The anthropometric measurements chosen for the study were the thigh circumference [50] at mid-thigh, and the volume of the knee extensors [51] for both lower limbs. Additionally, height and weight were measured using a precision scale.

The volume of the knee extensor muscles was estimated from thigh length and muscle thickness, following the method proposed by Miyatani et al. [52] As described by Miyatani, thigh length was measured as the distance between the greater trochanter of the femur and the lateral articular cleft of the knee. Thigh length and mid-thigh circumference were measured with a tape measure. Muscle thickness was measured at mid-thigh using an ultrasound transducer scanning head (Esaote MyLab™30, equipped with a 10 MHz linear-array ultrasound transducer LA523).

The procedure, as described in full in section 3 of the Supplementary Materials, was performed on both lower limbs, with the first measured limb randomly assigned.

### Force measurements

Strength and muscle activation testing were performed using a Cybex Humac Norm 2014 isokinetic dynamometer (CSMi Computer Sports Medicine, Inc., Stoughton, MA, USA). Isometric tests were conducted with the participant sitting upright on the dynamometer, with the hip flexed at approximately 90° and the knee held at 40° flexion (0° = full extension). The position of 40° of knee flexion was selected based on previous research from our laboratory on healthy controls [53], providing a normative reference for comparison with the current patient dataset. Furthermore, evidence from Krishnan and Theuerkauf suggests that testing at approximately 45° may maximise the detection of VA asymmetries between the reconstructed and non-reconstructed limbs following ACL injury [54]. 

### Voluntary activation

Measurement of VA is performed following the principle that a painless electric shock delivered to the motor nerve or the muscle during contraction should not generate extra-force if the voluntary activation is recruiting all fibres at their tetanic discharge frequency. In case of sub-maximal contraction, a transient increase of force (“twitch”) can be observed, with an amplitude inversely proportional to VA [55]. In the present study, the “interpolated twitch technique” (ITT) was adopted. This implies the stimulation, through large cutaneous electrodes, of a large portion of the quadriceps, and the delivery of a “doublet” of electric shocks (square waves; approximately 300–600 mA and 0.05–0.1 ms each, with a 10 ms interval) to the quadriceps. A first doublet is delivered at rest. After 3–5 s, a maximal voluntary contraction is requested. When a steady plateau can be visually appreciated in the joint moment tracings, a second doublet is delivered. The resting twitch was delivered before muscle contraction to avoid post-activation potentiation and the de-potentiation possibly induced by fatigue [56, 57]. The “twitch-like” increments in the recorded joint torque, i.e., the amplitude of the two “interpolated twitches” occurring at rest (“resting twitch”) and during voluntary contraction (“active twitch”), are used to measure the VA of the muscle (see below) [58]. 

During testing, starting with the healthy side, two repetitions of isometric maximal voluntary contraction (MVC) were performed for each limb, with a 3-minute break between the two repetitions. During each repetition, electrical stimulation was delivered at rest and then during MVC. During all MVCs, the patient was instructed to extend the knee “as hard and fast as possible,” a method reported to produce optimal results for lower limb isometric MVC in ACL-operated athletes [59, 60]. Participants received verbal encouragement.

Full details on instruments and settings for isometric testing and ITT are provided in Section 4 of the Supplementary Materials.

### Neurophysiological assessment

During the neurophysiological assessment, the participant sat on a sanitary armchair with armrests and a headrest.

Surface electrodes (15 × 20 mm NeuroTab disposable autoadhesive electrodes, Spes medica, Genova, Italy) were used for recording the EMG activity of the lower limb’s muscles (NeuroMepMicro portable EMG, 2009). EMG signals were sampled at 25,000 Hz using a 5–10,000 Hz passband [61], implemented as a first‑order recursive Butterworth high‑pass filter and a non‑recursive low‑pass filter; an adaptive non‑recursive notch filter removed 50 Hz power‑line interference and its harmonics.

The neurophysiological assessment began with the measurement of the compound muscle action potential (cMAP) in the VM and TA muscles of both lower limbs. For recording the cMAP of VM, the femoral nerve was stimulated in the region of the inguinal ligament, while for TA, the fibular nerve was stimulated in correspondence with the fibular head [62]. Recording electrodes were placed in a belly–tendon arrangement, and the nerves were activated electrically by percutaneous supramaximal stimulation to achieve the maximal cMAP [63]. 

Afterwards, single-pulse and paired-pulse TMS testing was performed. Single and paired-pulse TMS was performed using a figure-of-eight coil (model FEC-02-100, external diameter of each coil 100 mm) connected to a monophasic Neuro-MS/D Magnetic Stimulator (Neurosoft Ltd., Ivanovo, Russia; max peak field intensity 2.0 T). For VM and TA of both lower limbs, the resting motor threshold (rMT), recruitment curves of MEPs, and SICI were assessed.

For TMS testing, the TA muscle was preferred over other muscles of the lower extremity because previous evidence has suggested that the TA may be affected by ACL injury (e.g., TA showed increased volume on the ACL-injured limb in patients with the most impairments after ACL injury [64]). In addition, distal lower-limb muscles, such as the TA, are more accessible to neurophysiological testing, and including such a muscle in the investigation can serve as a benchmark, enhancing confidence in the reliability of the overall experimental framework.

Then, the H-reflex of the Soleus muscle was tested for both limbs. The rationale for this choice mirrored that of the TA: to investigate potential neuroplasticity in distal synergists using a technically accessible benchmark. The H-reflex examination was the only one conducted in a prone position.

It is worth noting that this study originally planned to perform brain mapping of the VM using TMS as well. However, due to the long time already required from the participants (also leading to a large number of eligible participants declining enrollment), this additional assessment was not performed.

#### Measures of corticospinal excitability

Measures of corticomotor excitability included the rMT (at rest) and the recruitment curve during submaximal contraction. These two measures are both influenced by changes in excitability at spinal and supraspinal sites, but are likely determined by different neural mechanisms [65]. 

Following rMT assessment, recruitment curves (i.e., input-output curves) were generated to characterise the relationship between stimulation intensity and MEP amplitude [65, 66]. Single-pulse TMS was delivered at varying intensities according to a systematic protocol (detailed in Section 5 of the Supplementary Materials). Crucially, the order of the stimulation intensities was randomised to minimise order effects. Four MEPs were recorded for each intensity step.

Recruitment curves were initially acquired under both resting and active conditions. However, the present analysis is restricted to the active recruitment curves. This decision was driven by the observation that, in 15 of the 20 investigated VM muscles (10 participants × 2 sides), the resting recruitment curve failed to reach a saturation plateau at the maximum stimulator output, thereby precluding a reliable fit to a sigmoidal function.

Assessing recruitment curves under active conditions fully aligns with literature recommendations, particularly given the well-known technical challenges in eliciting reliable MEPs from proximal lower limb muscles at rest [25, 33, 67–69]. 

For both VM and TA, the requested degree of muscle contraction was 20% of the one recorded during maximal voluntary contraction against resistance [67]. 

The entire procedure was repeated for the four muscles (VM and TA on both sides) in random order.

#### Measures of intracortical inhibition

Paired-pulse TMS was employed: a sub-threshold stimulus is used to condition the EMG response to a suprathreshold test stimulus [70]. Short-Interval Intracortical Inhibition (SICI) reflects the activity of intracortical inhibitory circuits, primarily mediated by GABA-A receptor-dependent interneurons. In this paired-pulse protocol, a subthreshold conditioning stimulus recruits low-threshold inhibitory interneurons that attenuate the corticospinal volley evoked by the subsequent suprathreshold test stimulus. Consequently, a lower amplitude of the conditioned MEP (relative to the unconditioned test stimulus) indicates stronger intracortical inhibition. From a measurement perspective, SICI is quantified as the ratio of the conditioned MEP to the unconditioned test MEP; therefore, a smaller ratio reflects greater inhibition (i.e., stronger SICI). To test SICI for lower limb muscles at rest, following the method described by Perez et al. [71], the conditioning stimulation was set at 70% rMT, and the test stimulus at 120% rMT, with an interstimulus interval of 2.5 ms to maximise inhibition [72]. Twenty stimulations, including ten conditioned and ten unconditioned stimulations in a randomised sequence, were delivered for each muscle (TA and VM on both sides) during resting. The order of the tested muscles was randomised.

#### Measures of spinal excitability

The H-reflex was used as an index of lower limb spinal excitability [73, 74]. The active electrode was placed on the Soleus muscle, and the reference electrode was placed over the posterior calcaneus. The H-reflex was evoked by stimulating the tibial nerve at the popliteal fossa with increasing electrical currents (in 1 mA steps, stimulus duration of 1 ms) while the participant was lying prone. The ground electrode was located between the active electrode and the stimulator. The H-reflex was assessed at rest.

### Ethics

This multicentric observational cross-sectional study (ClinicalTrials.gov: NCT04837417) complied with the Declaration of Helsinki and was approved by the ethical committee of the IRCCS Istituto Auxologico Italiano and by the Comitato Etico Milano Area 2 (AQUARIUS project, Ricerca Corrente IRCCS).

### Data and signal analysis

#### Estimates of quadriceps volume

Three muscle thickness ultrasound measurements were performed for each thigh and averaged.

The volume of the knee extensors was estimated using the equation provided by Miyatani [52]:

$$\:MV=(320.6\times\:T)+(110.9\times\:L)\:-4437.9$$

Where MV is the estimate of muscle volume in cm³, T is the average muscle thickness in cm, and L is the measurement of thigh length in cm.

By approximating the shape of the quadriceps to that of a cylinder, the cross-sectional area (CSA) of the quadriceps was calculated by dividing the estimated volume by the measured L.

#### Measuring quadriceps mean torque and rate of force development

The gravity-corrected torque signals were offline filtered (low-pass FIR filter, 1.3 Hz). In each isometric curve, the absolute mean torque was calculated in the 300 ms preceding the active twitch, after verifying that the torque level was stable on visual inspection of the tracings. Of the two MVCs recorded for each lower limb, the one characterised by the highest absolute mean torque was kept for analysis (maximum absolute mean torque). The maximum normalised mean torque was calculated for each lower limb by dividing the absolute mean torque by the CSA.

The RFD of the knee extensors’ torque was calculated using the slope of the torque-time curve. There is currently no univocal recommendation for the optimal threshold for detecting torque onset in RFD calculations [75]. In the present study, following previous research on both healthy individuals and ACLR patients [76–78], the time point at which the torque curve exceeded the baseline by 7.5 Nm was defined as the onset. The RFD in the first 50 ms from onset was selected as a measure of rapid force production. At the level of the muscle, this early RFD is believed to be influenced by the proportion of type II fibres [22, 79]. 

To parallel the analysis of recruitment curves from MEPs (see later), for each torque-time curve, torque increments were measured for each 10-ms time period from the onset to 50 ms after onset. Therefore, for each limb, five torque increments, measured at 10-ms intervals, were entered into the analysis.

#### Measuring quadriceps voluntary activation

To quantify the amplitude of both resting and superimposed (active) twitches, linear regression was applied to the torque data over a 300-ms window preceding stimulation. This model was used to predict the theoretical baseline torque at the exact instant of peak twitch tension. Consequently, twitch amplitude was calculated as the difference between the observed peak torque and the predicted baseline value. The VA was determined according to the following Eq. (53):

$$\text{VA= 1 - }\frac{\text{Active twitch}}{\text{Resting twitch}}{\%}$$

VA is a percentage, with 100% representing full voluntary muscle activation.

#### Analysis of recruitment curves

For recruitment curves and SICI, the MEP’s peak-to-peak amplitude was manually measured on each curve. In each participant, all resting (SICI) and activated (recruitment curves) MEPs were normalised to that muscle’s cMAP.

For each muscle recruitment curve, the mean value of normalised MEP amplitudes was calculated across all the stimulation intensities tested. Then, the highest mean value was defined as the maximum mean MEP for that recruitment curve.

We then investigated in greater detail how MEP amplitude varied with stimulation intensity. The central portion of the recruitment curve (the ascending ramp) is characterised by the steeper relationship between response (y-axis) and stimulus (x-axis). In the ascending ramp, which corresponds to about the middle 64% of the response range of the sigmoid-shaped curve [80], the relationship between MEP amplitude and stimulation intensity can be approximated as linear.

**Fig. 1:**
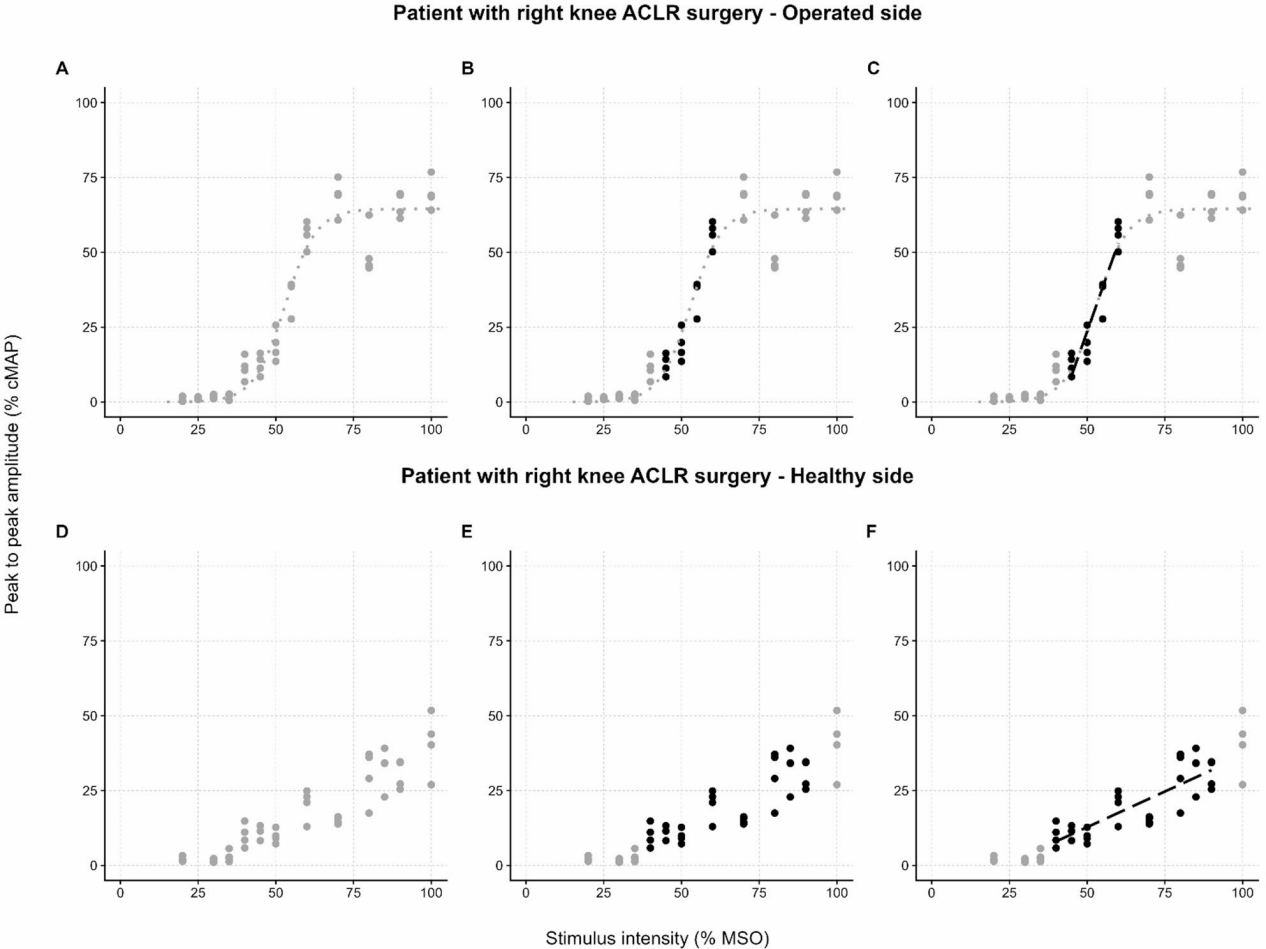
Recruitment curves of motor evoked potentials. In the recruitment curves, normalised MEP amplitudes (y-axis) with respect to stimulation intensities (x-axis) for the VM muscles of the operated (panels **A**, **B**, **C**) and healthy (panels **D**, **E,** **F**) sides of a right knee ACLR patient are reported. In the graphs, the procedure for selecting the MEP data that were eventually included in the statistical analysis of recruitment curves is illustrated. The leftmost panels (**A** and **D**) display all the MEP data collected for the recruitment curves of the operated (**A**) and healthy (**D**) side VM muscles, represented by grey circles. For graphical reference, panels **A** to **C** also show the Boltzmann three-parameter logistic function [65] fitted to the MEP data (grey dotted line). In the central panels (**B** and **E**), the stimulation intensities that provide MEPs with mean amplitudes ranging from 18% to 82% of the maximum mean MEP are represented as black circles. As described in the main text, these data were considered to correspond to the middle 64% of the response range (the linear portion of the recruitment curve) and were included in the statistical analysis. In the rightmost graphs (panels **C** and **F**), for graphical purposes, a linear regression line (black dashed line) has been fitted to the black circles to represent the steeper ascending portion of the sigmoid curve. The reader can also notice that, in the graphs displaying data from the healthy side VM muscle (panels **D**, **E**, **F**), MEP amplitudes continue to rise with increasing stimulation intensities, and no clear upper plateau can be identified. For this reason, a three-parameter logistic function was not fitted to these data

To identify the ascending ramp of the recruitment curve, the following procedure has been followed for each curve: (a) MEPs were plotted with stimulation intensity on the abscissa and normalised MEP amplitude on the ordinate; (b) the stimulation intensities providing MEPs with mean amplitudes comprised between 18% and 82% of the maximum mean MEP were identified; (c) in the subsequent statistical analysis, only these MEPs (corresponding to the middle 64% of the response range) have been considered. Figure 1 illustrates the procedure in detail.

In Fig. 1, panels A to C, the recruitment curve of the operated-side VM muscle from an exemplary participant is shown. As a graphical reference, in Fig. 1A, MEP data (grey circles) have been fitted (grey dotted line) with a Boltzmann three-parameter logistic function (as described in [65, 81]). Data points corresponding to stimulation intensities that yield mean MEPs with amplitudes between 18% and 82% of the maximum mean MEP (i.e., the central steeper part of the recruitment curve) are identified (black circles in Fig. 1B). For graphical purposes only, a linear regression line (black dashed line) has been fitted to the black circles (Fig. 1C). The regression line roughly overlaps the central portion of the sigmoid-shaped recruitment curve, as expected.

In some instances, even during voluntary muscle activation, the recruitment curves lacked a distinct upper plateau, showing a monotonic increasing trend. Figure 1, panels D to F, illustrates such a case by reporting MEP data of the non-operated side VM muscle of the same exemplary participant. The reader can see that, in Fig. 1D, for the same stimulation intensity shown in panels A-C, the amplitude of MEPs increases proportionally to the stimulation intensity. In any case, between the 18% and 82% intensity boundaries (black circles in Fig. 1E and F), the regression line (black dashed line in Fig. 1F) appears to be a good approximation of the data. Thus, only these MEPs have been considered in the analysis, following the same procedure described above.

As previously noted, the absence of a distinct upper plateau was also frequently observed in resting recruitment curves. However, an additional critical factor necessitating their exclusion was the sparsity of the recruitment. At rest, responses were often elicited only at a few high intensities. This lack of data distribution rendered the calculation of the intensities corresponding to 18% and 82% of the maximum mean MEP often collapsed into a single intensity value or could not be reliably determined.

#### Measurement of Hmax/Mmax ratio from H-reflex curves

For each stimulation intensity of the H-reflex curve, the peak-to-peak amplitudes of the M-wave and H-reflex were measured. For each limb, the Hmax/Mmax was calculated as the ratio between the amplitudes of the maximal H-reflex and maximal M-wave.

### Statistics

The exact Wilcoxon signed-rank test for paired samples (using the Pratt method for handling ties [82]) was used to compare the MV of knee extensors, the thigh circumference, the maximum normalised mean torque and VA of knee extensors, the rMT of TA and VM, the maximum mean MEP of TA and VM, and the Hmax/Mmax ratio between the operated and non-operated limbs.

The outcome variables were reported as means (SD), except when stated otherwise.

For non-parametric data, the effect size (r) was calculated as the ratio of the Z statistic (yielded by the test) to the square root of the total sample size (N). Following Cohen’s benchmarks, effect sizes were interpreted as small (0.1 ≤ *r* < 0.3), moderate (0.3 ≤ *r* < 0.5), and large (≥ 0.5) [83]. 

In contrast to the measures reported above, which yielded a single observation per condition (e.g., one circumference value per limb), the recruitment curve and SICI datasets were organised into multilevel hierarchies. In this structure, clusters of MEPs were nested within specific muscles (TA or VM; operated vs. non-operated limb) and subsequently within each of the ten patients. The database of RFD values showed a similar structure. Therefore, the hierarchical organisation of data and the violation of the assumption of independence were addressed by using linear mixed-effects models (LMM) [84] for the statistical analysis of recruitment curves, SICI, and RFD (random intercept and random slopes model) [85].

The model equation was:

$$\begin{array}{lc}Data\,value=&\beta_0+\\&\beta_{1\;}Side+\\&\beta_2\;Parameter+\\&\beta_3\left(Side\times Parameter\right)\;+\\&Random\;+\\&\epsilon\end{array}$$

Where:

- *Data value* (i.e., the dependent variable) is the normalised MEP amplitude for recruitment curves and SICI, or the torque value for RFD.
- *β* represents fixed effects coefficients.
- *Side* is an independent variable referring to either of the two lower limbs (operated vs. non-operated limb; categorical variable).
- *Parameter* is an independent variable that (a) in the analysis of recruitment curves, refers to the stimulation intensity (% MSO; continuous variable), (b) in the analysis of SICI, corresponds to the trial condition (conditioned vs. unconditioned stimulations; categorical variable), and (c) in the analysis of RFD, corresponds to the time point of a given torque value measured from the onset (ms; continuous variable).
- *Side × Parameter* refers to the interaction between *Side* and *Parameter*.
- *Random* refers to the random effects.
- *ε* represents the model’s residuals.

The model’s random structure included intercepts (for patients) and slopes (for limb side). Thus, the model assumes that a certain intercept characterises each patient and allows each patient to behave differently in response to the effect *Side*; the effect of *Parameter* is fixed.

For hypothesis testing, a Type III ANOVA was conducted to assess the statistical significance of the fixed effects in the regression models. Degrees of freedom were calculated using the Satterthwaite approximation.

Regarding the other regression assumptions, the normality of the residuals and the homogeneity of their variance were visually verified. When necessary, to satisfy these assumptions, the response variable was log-transformed or square-root transformed, and hypothesis testing was conducted on the transformed data. In all cases, for clarity, data are reported untransformed in the graphs.

If the assumptions of homogeneity of variance and normality of the residuals were not met by variable transformation, the LMM results were double-checked using a non-parametric approach, i.e., aligned-ranks transformation ANOVA (ARTanova) [86]. 

The type 1 error probability was set at α = 0.05. The Benjamini-Hochberg Procedure was used to correct for multiple comparisons [87, 88]. 

Signal software (version 8, Cambridge Electronic Design Ltd, Milton, Cambridge, England) was used to analyse torque signals. NeuroMEP.NET software (version 4; Neurosoft, Ivanovo, Russia) was used to analyse the cMAP and MEP signals. All the statistical analyses were run in R (version 4) [89]. R was used for plotting and editing figures.

## Results

### Screening and recruitment

Between 2020 and 2023, a total of 1,429 patients underwent ACLR at the recruiting orthopaedic Institute. According to the inclusion and exclusion criteria, 1347 patients were excluded based on the information reported in the clinical records (e.g., sex, age, surgical technique, BMI, additional surgical procedures, relevant knee axial deviations, and medical co-morbidities). Seventeen additional patients were excluded after a telephone conversation revealed the study’s criteria were not met. A total of 65 patients were eligible for recruitment. Fifty patients refused to participate because they were not interested. One recruited participant had unintentionally concealed an exclusion criterion and was excluded after the clinical examination but before the instrumental assessments were performed; one patient developed flu-like symptoms just before starting the examination and was dropped; three recruited patients participated in one of the assessment sessions and then dropped out because they deemed the time required by the study too demanding. Ten patients completed all testing sessions and were considered in the following analysis. In the final sample, the median [interquartile range] interval between the dynamometric and neurophysiological tests was 6 [6.75] days.

### Participants

Six of the ten recruited patients had ACLR on the right limb, with the remaining four operated on the left side. The mean time from surgery was 258 days (approximately 8 months), with a range of 188–371 days (6–12 months). Details on the ten participants are provided in Table 1.

No adverse events were observed during testing.

**Table 1:** Participants. Characteristics of the ten ACLR patients recruited in the study. ID: patient identification code; Days from surgery: number of days since ACLR; BMI: Body Mass Index (kg/m^2^); Operated side: right (R) or left (L) ACLR

ID	Age	Days from surgery		BMI		Operated side
1	31	195		23.4		R
2	29	329		22.2		R
3	20	188		19.7		R
4	30	244		23.9		R
5	27	371		20.9		L
6	30	235		22.4		L
7	25	264		22.6		R
8	28	193		24.6		R
9	20	290		24.7		L
10	20	271		24.6		L
Mean	26.0	258		22.9		
SD	4.47	60.4		1.68		

### Anthropometric measurements

For estimates of MV, results from the Wilcoxon signed-rank test showed a significant difference (Z = -2.55, *p* = 0.035) between the two limbs, with MV of the non-operated side (mean ± SD = 2264.6 ± 345.1 cm^3^) being about 8% higher than the operated side (2082.9 ± 386.2 cm^3^).

A significant difference (Z = -2.76, *p* = 0.035) was also detected for thigh circumference, with the non-operated limb showing a 4% greater mean circumference (52.2 ± 2.7 cm) than the operated one (50.2 ± 3.3 cm).

### Knee extensors’ torque, rate of force development, and voluntary activation during maximal isometric contraction

Neither the maximum normalised mean torque (Z = 1.38, *p* = 0.435) nor the VA of the knee extensors (Z = 0.46, *p* = 0.922) was significantly different between the two limbs. The torque, normalised with respect to the CSA, was 2.97 ± 0.48 Nm cm^− 2^ for the non-operated limb and 3.14 ± 0.7 Nm cm^− 2^ for the operated side. The VA, measured with the ITT, was 87.2 ± 6.9% for the non-operated limb and 87.4 ± 8.9% for the operated one.

For RFD, the assumptions of homogeneity of variance and normally distributed residuals were not fully complied with by transforming the dependent variable. Therefore, results from LMM were double-checked using aligned ranks transformation ANOVA (ARTanova) [86, 90]. A significant relationship was found between RFD and time from onset (F (1, 80) = 314.59, *p* < 0.001). No significant relationships were found with respect to the side (F (1, 24) = 0.06, *p* = 0.770), while a significant interaction was found between side and time from onset (F (1, 80) = 5.30, *p* = 0.026).

**Fig. 2:**
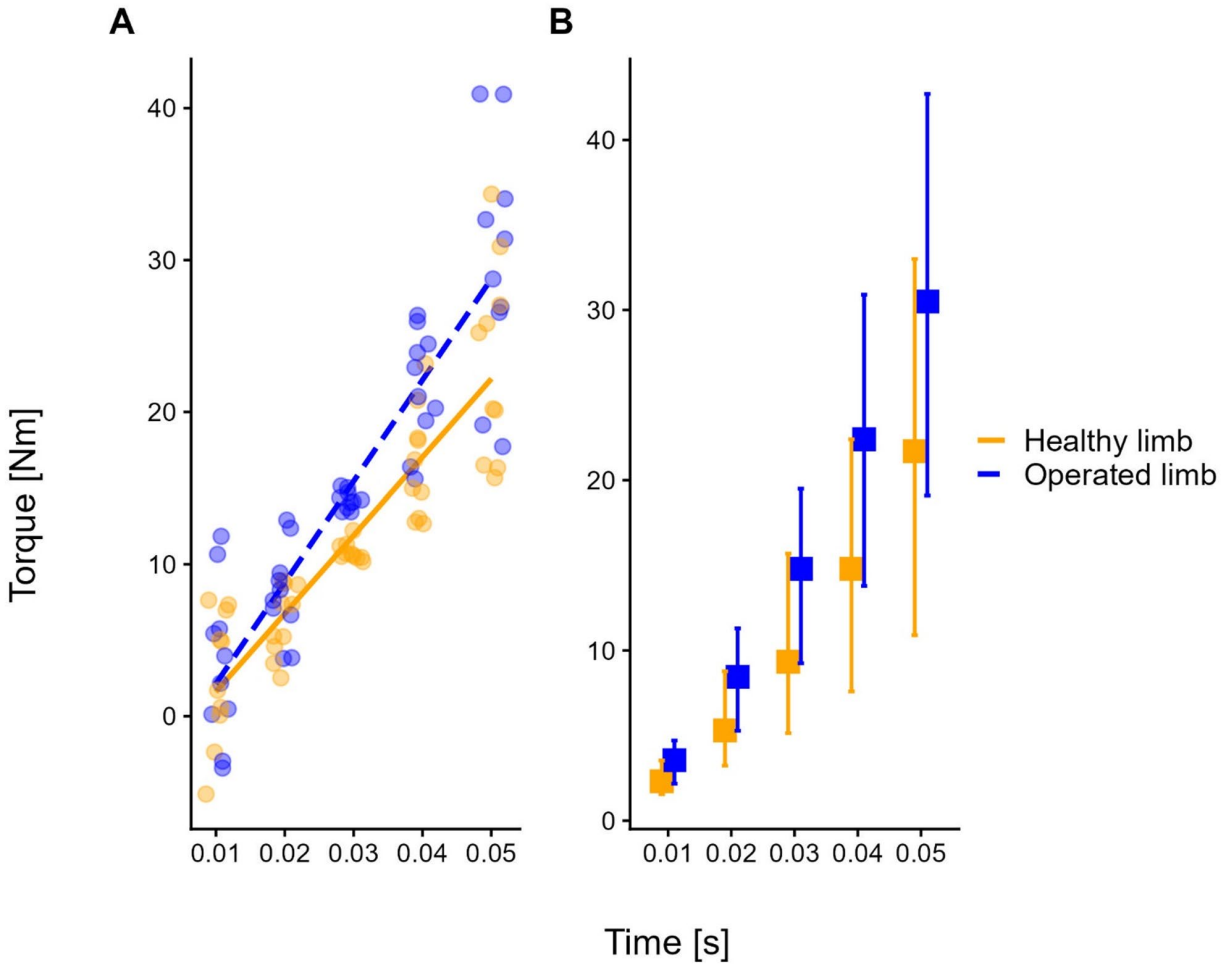
Rate of force development (0–50 ms) during maximal isometric knee extension. The graphs illustrate the increments in knee extensor torque (y-axis) over time from the onset (x-axis) for the ACL-reconstructed (blue) and healthy (orange) limbs. In Figure 2A, regression lines with partial residuals [91], derived from linear mixed-effects models that include the time from onset (0 to 50 ms, in 10-ms steps) and limb side (operated vs. healthy limb) as predictors, are displayed. Partial residuals are jittered to improve clarity. The regression slope was significantly steeper on the operated side (blue dashed line in panel **A**), compared to the healthy side (orange continuous line in panel **A**). Figure 2B shows the median values and 95% confidence intervals of torque increments in the 0-to-10 ms, 10-to-20 ms, 20-to-30 ms, 30-to-40 ms, and 40-to-50 ms time intervals for the operated (blue) and healthy (orange) sides

Figure 2 illustrates the relationship between torque increment and time from onset for the operated and healthy sides. In Fig. 2A, regression lines from LMM show that the slope of RFD of the operated limb was steeper than that of the sound one, thus indicating an increased ability to increase torque in the first 50 ms of muscle contraction. Figure 2B shows the median values and 95% confidence intervals of torque increments in the 0-to-10 ms, 10-to-20 ms, 20-to-30 ms, 30-to-40 ms, and 40-to-50 ms time intervals.

### Measures of corticospinal excitability

No difference was found for the rMT of the TA (Z = -0.21, *p* = 0.922) and VM (Z = 0.31, *p* = 0.922) muscles. Mean ± SD values of rMT were 60.5 ± 12.9% and 59.8 ± 16.2% for the non-operated and operated side TA, respectively, while they were 69.5 ± 11.3% and 68.9 ± 14.4% for the non-operated and operated side VM, respectively.

No difference was found for the maximum mean MEP of the TA (Z = -0.26, *p* = 0.922) and VM (Z = 1.48, *p* = 0.435) muscles. Mean ± SD values for VM maximum mean MEP were 35.1 ± 14.0% and 53.4 ± 36.4% for the non-operated and operated sides, respectively. For the TA maximum mean MEP, mean ± SD values were 63.9 ± 15.4% for the non-operated side and 62.6 ± 19.5% for the operated one.

In the analysis of the TA recruitment curves, a significant relationship was found between MEP amplitude and stimulation intensity (F(1, 245) = 322.2, *p* < 0.001). No significant relationships were found with respect to side (F (1, 64) = 3.36, *p* = 0.072) and the side-intensity interaction (F (1, 101) = 3.31, *p* = 0.072).

For the VM recruitment curves, a significant relationship was found between MEP amplitude and stimulation intensity (F(1, 295) = 273.56, *p* < 0.001) in the VM muscle. No significant relationship was found for the side of the limb (F (1, 42) = 2.30, *p* = 0.137). For VM, a significant interaction was found between the limb side and stimulation intensity (F(1, 272) = 12.35, *p* = 0.001).

**Fig. 3:**
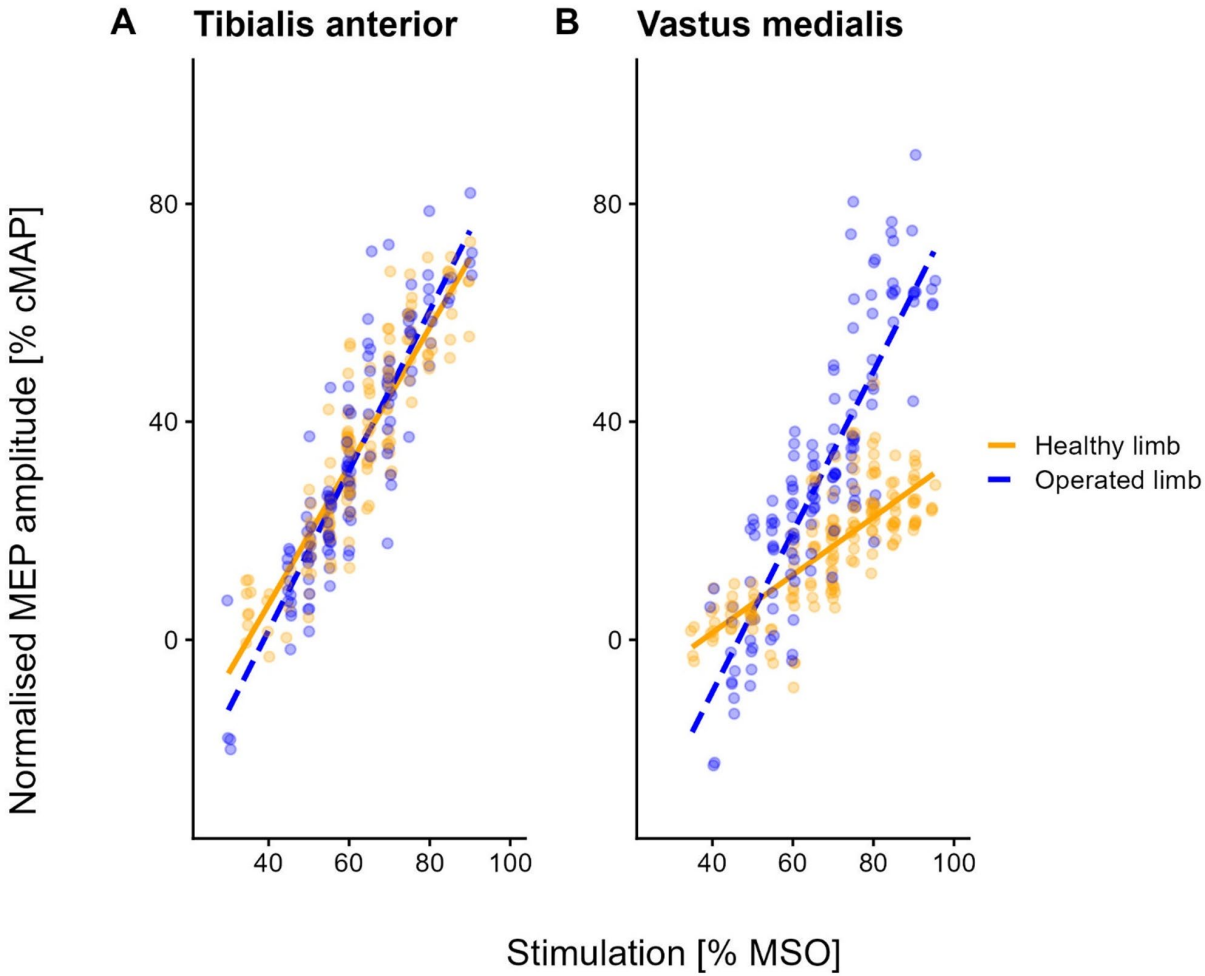
Relationship between MEP amplitude and stimulation intensity for the Vastus medialis and Tibialis anterior muscles. The graphs illustrate the modifications in normalised MEP amplitude (y-axis) with increasing stimulation intensity (x-axis) for the operated (blue) and healthy (orange) Tibialis anterior (panel **A**) and Vastus medialis (panel **B**) muscles. Regression lines with partial residuals [91], derived from linear mixed-effects models that include stimulation intensity and limb side (operated vs. healthy) as predictors, are displayed. Partial residuals are jittered to improve clarity. For both the operated and healthy limb, the higher the stimulation intensity, the higher the MEP amplitudes of the Tibialis anterior and Vastus medialis. However, for the Vastus medialis muscle, the regression slope was significantly steeper on the operated side (blue dashed line in panel **B**), compared to the healthy side (orange continuous line in panel **B**). It implies that, with increasing stimulation intensities, the amplitude of MEPs recorded from Vastus medialis increased faster on the operated side. Conversely, no significant inter-limb difference was found for the Tibialis anterior muscle. For the TA muscle, stimulation intensities ranged from 30% MSO to 90% MSO in 5% MSO increments. For the VM muscle, stimulation intensities ranged from 35% MSO to 95% MSO in 5% MSO

These patterns are visualised in Fig. 3. While recruitment profiles for the TA were comparable between limbs (Fig. 3A), for the VM, the operated side exhibited a steeper slope (i.e., greater gain) than the healthy limb (Fig. 3B).

### Measures of intracortical inhibition

In the analysis of SICI, a significant difference was found between conditions (i.e., conditioned vs. unconditioned stimulations) for both TA (F(1, 378) = 275.74, *p* < 0.001) and VM (F(1, 366) = 332.63, *p* < 0.001). No difference was found in the comparison between the two limbs for both TA (F (1, 10) = 4.38, *p* = 0.063) and VM (F (1, 10) = 0.80, *p* = 0.394). Likewise, no difference was found for the interaction between side and condition for both TA (F(1, 378) = 0.02, *p* = 0.892) and VM (F(1, 366) = 0.36, *p* = 0.550).

**Fig. 4:**
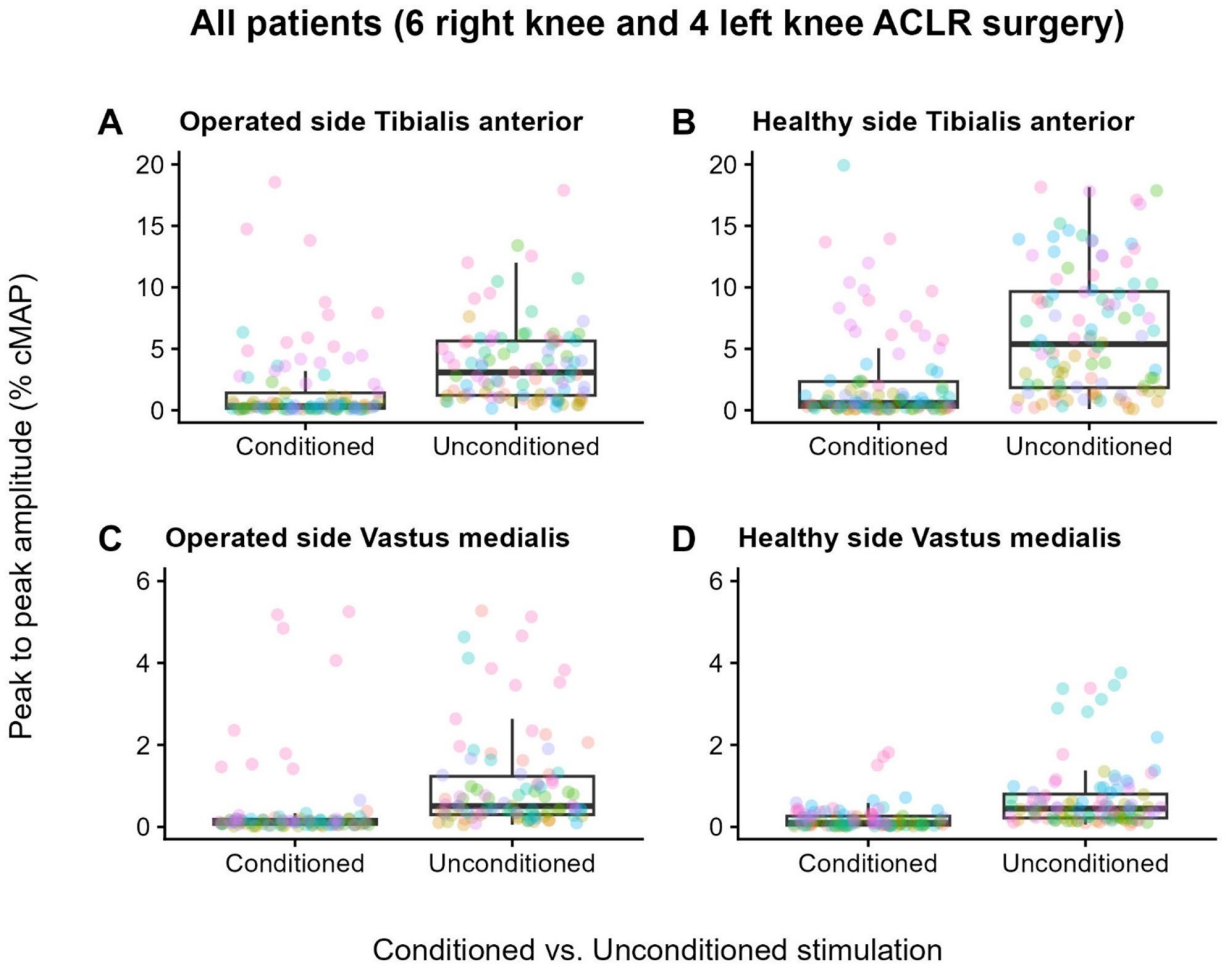
Short-interval intracortical inhibition of the Vastus medialis and Tibialis anterior muscles. In the graphs, box plots of MEP amplitudes of conditioned and unconditioned trials from SICI testing are displayed for the Tibialis anterior (upper row) and Vastus medialis (lower row) muscles of the operated (panels **A** and **C**) and healthy (panels **B** and **D**) limbs of the entire sample of ten patients with ACLR. In box plots, the box spans from the first to the third quartile, with a line representing the median, while the upper and lower whiskers extend to the furthest data point that is within 1.5 times the interquartile range. In each box plot, individual data points are represented as coloured circles (each colour denotes a different participant; circles are jittered to improve clarity). As expected, the median MEP amplitude is lower in conditioned trials than in unconditioned trials across all investigated muscles. No significant inter-limb differences were found.

Figure 4 shows box plots of normalised MEP amplitudes for conditioned and unconditioned stimulation on the operated (panels A and C) and healthy (panels B and D) sides of the entire sample of 10 patients. The reduction in median MEP amplitudes in conditioned trials relative to unconditioned (i.e., control) stimulations is SICI. No differences in the SICI-induced reduction of MEP amplitude were found between sides.

### Measures of spinal excitability

The Soleus Hmax/Mmax ratio was not different (Z = -0.153, *p* = 0.922) between the non-operated (0.39 ± 0.24) and the operated (0.38 ± 0.26) sides.

Effect sizes and 95% confidence intervals for all single-point measures (e.g., anthropometry, MVC, TMS thresholds) are reported in Sect. 7 of the Supplementary Materials. LMM slope estimates for the dynamic analyses (RFD, recruitment curves, and SICI) are detailed in Sect. 8.

## Discussion

Results have shown that patients experienced significant thigh muscle atrophy on the operated side. Although the difference in the maximum mean MEP did not statistically differ between the healthy and operated limbs for both TA and VM, LMM demonstrated that the relationship between MEP amplitude and stimulation intensity was steeper for the VM muscle of the operated side compared to the sound limb (Fig. 3B). Likewise, an increased RFD has been found for the knee extensors of the operated limb during maximal explosive isometric contractions, as indicated by the steeper slope of the torque-time curve (Fig. 2A). No inter-limb differences have been observed for quadriceps maximum normalised torque and for measures of rMT and muscle activation. Also, the intracortical excitability, as measured by the SICI for both TA and VM, and the spinal reflex excitability, as indicated by the Hmax/Mmax ratio of the Soleus muscle, did not show significant differences in the comparison between the operated and non-operated limb.

Quadriceps muscle atrophy is a common finding after ACL rupture and ACLR [51]. For example, in a sample of 70 patients evaluated 6–12 months after ACLR with semitendinosus-gracilis autograft, Konishi et al. reported an average side-to-side deficit of 7% in total quadriceps volume, as measured with magnetic resonance imaging [92]. In the present study, MV was estimated from ultrasound measures of muscle thickness using a validated method [52] and yielded superimposable results (an 8% inter-limb difference in MV). This method primarily considers the components of the Rectus femoris and Vastus intermedius in the computation of muscle thickness (see section 3 of the Supplementary Materials), and our results align with previous evidence from Yang et al., who have detailed with ultrasound the selective involvement of the four components of the quadriceps after ACLR, reporting a severe reduction in thickness affecting especially the Vastus intermedius in the early days after surgery [12]. However, the origin of the atrophy observed in ACL-injured and reconstructed patients remains unknown, with multiple mechanisms suspected to contribute [93]. Quadriceps atrophy has usually been interpreted as a direct consequence of disuse (due to immobilisation or decreased exercise load) or of arthrogenic muscle inhibition [94], i.e., the diminished efferent motor drive to the muscles, which results from the altered afferences originating from the injured joint. More recently, the roles of mitochondrial autophagy [95] and of atrophy-inducing cytokines and hormonal factors have been hypothesised [93]. Additionally, it is unclear whether and how decreased muscle mass contributes to quadriceps weakness and reduced activation observed in ACLR patients. Previous research investigating the relationship between quadriceps wasting and muscle performance has provided conflicting results. In general, the contribution of quadriceps VA and CSA, alone or together with other covariates, explains only a minor part (e.g., 30% [96] or 38% [97]) of the variance in knee extension strength, leading to the hypothesis that other factors, like changes in muscle architecture and mechanics (e.g., fascicles’ length and pennation angle [98]) or histological properties (e.g., fibre type), may be more relevant than the overall muscle volume [97]. From this standpoint, our results are consistent with prior literature: the asymmetry in muscle volume and thigh circumference was not accompanied by significant interlimb differences in isometric maximum normalised mean torque or VA. However, a limitation of this study is that, due to the cross-sectional design, we cannot conclude whether the alterations in quadriceps volume and circumference resulted from ACLR surgery, were present before surgery, or even before the ACL injury.

The fact that no inter-limb differences were detected for quadriceps VA was not unexpected. Previous research has shown that deficits in quadriceps VA occur after ACL injury and in ACLR patients, but these deficits have been reported to involve both the operated and the non-operated side, as revealed by comparisons between ACLR patients’ limbs and those of healthy controls. Notably, this evidence is not dependent on the technique used for measuring VA, as similar results have been reported in studies adopting either the ITT [99, 100], as done here, or the central activation ratio (CAR) [14, 101], an alternative method based on maximal electrical stimulation of the entire muscle [102]. 

Regarding the maximum normalised mean torque of the knee extensors, no significant differences were detected between sides. It is important to highlight that the mean torque, normalised to the quadriceps CSA, was higher on the operated side. Without normalisation, the maximum absolute torque was 152.5 ± 36.1 Nm and 148.3 ± 48.1 Nm for the non-operated and operated side, respectively. Again, this supports evidence that factors other than muscle volume alone may be more relevant to muscle strength (as previously discussed). This result may also be attributed to the study’s design, as strength testing was always performed on the sound limb first, so that a learning phenomenon favouring the operated side cannot be excluded. For completeness, absolute and normalised torque data for the sample of ten ACLR patients recruited here are reported in Sect. 6 of the Supplementary Materials.

The literature on ACLR reports bilateral deficits in maximal isometric quadriceps strength relative to controls that persist for years after surgery [14]. In this regard, another limitation of the present study is that we did not recruit a control group; only inter-limb comparisons were performed in our sample of ACLR patients. Therefore, although our data are consistent with previous evidence reporting the absence of significant interlimb asymmetries in VA and maximum torque in patients with unilateral ACLR, the study design does not allow us to compare patients’ performance with that of healthy individuals. However, the average maximum torque of ACLR patients was lower than that measured in a sample of healthy young men in a previous study from our group (184.85 ± 27.25 Nm and 168.99 ± 23.77 Nm for the dominant and non-dominant limbs, respectively [53]). Additionally, we didn’t examine inter-limb differences in function and morphology of the hamstring muscles, which are reported to be affected by ACLR [103]. These muscles, which prevent forward tibial shearing during knee extension, can be overactivated in subjects with ACL deficiency [104], thus decreasing the net extensor torque measured by the dynamometer. However, it is worth noting that in the present study, an oscilloscope was used throughout all isometric testing to monitor surface EMG of the Vastus lateralis and Biceps femoris muscles to verify the absence of relevant hamstrings activity, although these data were not recorded.

As already anticipated, it has recently been hypothesised that alterations in the central nervous system may cause poor recovery after ACLR surgery despite proper rehabilitation treatment. Indeed, a “cascade of neurophysiological alterations” has been reported to occur after ACL injury [105], and the injury to the knee has been regarded as a “neurophysiological dysfunction” [106] rather than just a local joint injury. Among the neurological factors potentially contributing to muscle atrophy and weakness, we can include reduced volitional recruitment of motor units (reflecting cortical reorganisation), less efficient muscle synergies, and inhibitory spinal reflexes [107]. 

As discussed for quadriceps maximum torque and VA, a recent meta-analysis has documented that even the cortical motor threshold does not show significant differences between the operated and non-operated side in ACLR patients, while higher motor thresholds (indicating reduced excitability) are present bilaterally in the quadriceps of ACLR patients when compared to healthy controls [23]. Accordingly, our study found no differences in the rMT of the TA and VM muscles between the two sides. However, we found that the increase in MEP amplitude with increasing stimulation intensities was steeper for the operated side VM (see Fig. 3B).

At first glance, the absence of a significant inter-limb difference in rMT may seem to contradict the asymmetry in the slope of VM. However, these parameters likely represent different measures of corticospinal excitability [108]. Stimulations at intensities near rMT recruit only the most excitable neurons within the motor cortex. With increasing stimulation intensities and in activated conditions (in this study, TMS was delivered during submaximal contractions to assess recruitment curves), even neurons that are less excitable or spatially farther from the centre of activation, as determined by the magnetic stimulus, are recruited [108]. Our results may thus imply that, although the initial drive needed to elicit responses is not different between the two hemispheres, the motor cortical representation could be larger in the hemisphere contralateral to the operated limb, or that the overall excitability could be greater once a large number of cortical neurons are activated [32]. Notably, a study by Ward et al. didn’t observe differences in quadriceps cortical motor representation in ACL-injured individuals [109], but we are not aware of similar studies following ACLR. Moreover, another study reported increased MEP amplitudes in the quadriceps muscle on the affected side in patients with chronic patellofemoral pain syndrome [110]. Further research is thus necessary to reach more definitive conclusions.

Our result also aligns with some recent neuroradiological findings. In general, cross-sectional neuroimaging studies have supported conclusions drawn from neurophysiological testing by providing additional evidence of cortical and cerebellar reorganisation in patients with ACLR [107, 111, 112]. More in detail, a functional magnetic resonance imaging study reported that ACLR patients exhibited increased activation in the primary motor cortex and lingual gyrus of the hemisphere contralateral to the operated knee and in the ipsilesional secondary somatosensory area, with diminished activation in the ipsilesional motor cortex and vermis, during unilateral knee flexion and extension motor tasks involving the operated limb, when compared to healthy matched controls [112]. This evidence has been interpreted as reflecting an increased need for cortical drive to engage the quadriceps following ACL injury and reconstruction [112]. It has also been speculated that the increased activation in the ipsilesional secondary somatosensory area may indicate bilateral neuroplasticity involving sensory processing. Accordingly, another study has reported that patients showing lower quadriceps maximal torque (i.e., a weaker quadriceps) in the operated limb (relative to the sound one) during isokinetic maximal voluntary contractions also displayed higher activity in the contralesional premotor cortex, lingual gyrus, and superior parietal lobule [107], again indicating a relatively increased neural demand to elicit quadriceps contractions.

This increased corticospinal excitability, which in our study involves only the VM muscle, may have different possible explanations. First, it may reflect a compensatory mechanism for quadriceps atrophy: corticospinal drive to the knee extensors would increase to maintain the required torque output. Notably, the Vastus intermedius, which is severely affected by muscle wasting, is a primary determinant of knee extensor torque [113]. Second, it may be a result of the therapeutic exercises the patient has undergone after surgery (and possibly even before surgery). Our sample was recruited, on average, 8 months after surgery, and all patients were still actively engaged in physical therapy programs aimed at restoring knee function. Third, it may be an effect of asymmetric training during sport practice, limb preference (i.e., footedness), or other factors that may influence cortical and corticospinal activity, which our study did not control for. However, as previously discussed, we cannot determine whether these alterations in corticospinal excitability resulted from the surgery or were present prior to it. Lastly, it must be acknowledged that MEP amplitude can exhibit high trial-to-trial variability, which may be due not only to experimental factors but also to biological ones [114]. Several precautions have been taken to improve the reliability of the MEP measures: as suggested by the literature, testing MEPs during submaximal muscle contractions, as performed here, reduces the variability of MEP amplitudes [115]; multiple MEPs have been recorded for each tested stimulation intensity; the order of the stimulation intensities tested for each limb has been randomised. Regarding the differences observed in VM corticospinal excitability between the reconstructed and sound limbs, we must recall that the present study’s design doesn’t allow us to compare the inter-limb differences observed in patients with the physiological inter-limb differences of healthy controls.

The increased corticospinal excitability toward the operated-side VM is consistent with the steeper RFD observed on the same side. In this study, we assessed RFD in the first 50 ms from onset. RFD measures the ability to recruit motor units maximally and rapidly. This early RFD depends on the maximal discharge rate of motor units, the speed of motor unit recruitment, and muscle fibre properties, such as size and fibre type [76]. Thus, the observed increase in corticospinal excitability (i.e., the steeper slope of the recruitment curve) may represent the mechanism facilitating an upregulated “neural drive”—i.e., a stronger descending volley generating rapid excitatory post-synaptic potentials (EPSP) summation at the motoneuron pool. This heightened neural drive would enable fast, time-compressed recruitment of high-threshold motor units (those contributing most to RFD), thereby compensating for the reduced muscle cross-sectional area (i.e., contractile mass). Consistent with our results, a higher cortical neural drive does not necessarily entail a higher peak force, as the rate of rise of muscle force and its maximal level can be controlled independently [116, 117] through frequency modulation of motoneuronal discharge from supraspinal inputs [118]. It is worth noting that ballistic-type contractions, like the ones tested here with the dynamometer, are characterised by high RFD and can display selective activations of large motor units (possibly bypassing the size principle of Henneman) and pre-activation of muscle fibres (i.e., activation of the involved muscles in preparation for the movement) [119]. Furthermore, this profile (high RFD, high excitability) is consistent with the specific adaptations induced by resistance strength training, which has been shown to increase RFD, possibly due to enhanced neural drive [120]. This opens the possibility of using measures of corticospinal excitability and joint torque production to assess the effects of therapeutic exercise following ACLR.

With respect to muscle histology, changes in skeletal muscle morphology and fibre types can also account for differences in RFD. Histological adaptations occur in the quadriceps muscle of patients with ACL injuries. A study by Noehren et al. reported selective atrophy of type-IIA fibres, increased extracellular matrix, and reduced satellite cells in the Vastus lateralis muscle of the affected side after ACL injury [121]. Interestingly, in that study, the alterations observed in muscle biopsies did not improve after ACLR and subsequent rehabilitation. To the contrary, a further loss in type-IIA fibres and an increase in type-IIA/X hybrid fibres were observed after surgery [121]. Such a shift toward faster fibre types, with an increased proportion of hybrid fibres, is similar to the pattern observed under conditions of disuse, including inactivity and injury [122]. However, exercise has been shown to alter the proportions of fibre types [122]. The functional role of the morphological changes that occur after ACL injury and ACLR remains debated, and future research should examine whether exercise interventions, particularly resistance training, may drive changes in the skeletal muscle phenotype of ACLR patients.

It is worth noting that the RFD result appears to contradict the meta-analysis by Turpeinen, which reported RFD deficits in the operated-side quadriceps after ACLR. However, as noted by the Authors, most studies included in the meta-analysis involved patients with bone-patella tendon-bone autografts, which may have affected the results [22]. 

In our study, SICI didn’t reveal differences in intracortical excitability between the two sides. This contrasts with the results of a recent meta-analysis, which reported a significant increase in intracortical inhibition in the hemisphere contralateral to the injured or reconstructed limb, indicating increased GABAergic activity on this side [23]. However, out of the three studies considered in the meta-analysis, the only one examining SICI at rest has shown results similar to ours [32], while the other two have assessed SICI during active muscle contraction [27, 109]. Therefore, methodological heterogeneity may account for the differing findings.

The study demonstrated increased corticospinal excitability from the contralesional motor cortex to the motoneurons of the knee extensors ipsilateral to the reconstructed ACL. This increase was inferred from the steeper slope of the VM recruitment curve on the operated side compared with the contralateral side. We acknowledge that the steeper slope may reflect factors other than increased excitability of the primary motor cortex. Specifically, upregulation of spinal premotoneuronal circuits that contribute to motoneuron recruitment could theoretically produce MEP changes identical to those observed here. However, this “spinal hypothesis” is made less likely by our H-reflex results, which showed no asymmetry. That said, a limitation must be noted: while the Soleus reflex provides a proxy for general spinal excitability of the lower limb, a more definitive exclusion of spinal contributions would have required H-reflex assessment directly from the VM.

Indeed, most of the literature has investigated the Hmax/Mmax ratio of the VM muscle, with meta-analyses reporting a bilateral increase in spinal excitability in ACLR patients when compared to healthy individuals [23]. Here, the H-reflex was recorded from the Soleus muscle both for technical feasibility and to test the hypothesis that changes in CNS plasticity would affect the entire limb in ACLR patients. In this regard, no inter-limb differences were found for either the Soleus H-reflex or the corticospinal excitability of the TA muscle. Thus, this hypothesis was not supported.

We have already mentioned the main limitations affecting our study (e.g., the absence of a control group and the cross-sectional design). In particular, a control group would allow comparison of the between-limb differences observed in ACLR patients with those observed in healthy individuals. It is also necessary to note the reduced sample size and the homogeneity of the recruited sample, as they may affect the generalisability of the results. An inevitable trade-off exists between sample homogeneity and the generalizability of the results. As previously discussed, sample homogeneity has been prioritised in this study. Although this sample size is small for clinical trials, we believe it is justified for this exploratory physiopathological study for three main reasons. First, we experimentally minimised sources of variance by applying highly stringent inclusion and exclusion criteria. In this regard, we deliberately prioritised homogeneity over sample size. Such rigorous selection is intended to minimise intersubject variability (i.e., biological noise), thereby enhancing the signal-to-noise ratio and enabling more robust detection of the effects, despite the sample size. Second, we adopted specific procedural safeguards to mitigate the inherent variability of TMS measures. As previously discussed, we assessed MEPs during submaximal voluntary contraction rather than at rest, marked the position of the hot spots on the participant’s skin to ensure consistent coil placement, and randomised stimulation intensities. Third, the statistical analysis of corticospinal excitability and SICI utilised LMMs rather than relying on a single mean value per subject. By modelling individual variability, LMMs enable more precise estimation of within-participant effects, thereby increasing sensitivity to detect changes in the response variable driven by the regressors. This exploratory study was also designed to identify outcomes worth considering in future larger clinical trials. Given the time required for the tests performed here, careful selection of the outcomes is mandatory. Indeed, the instrumental assessment had to be conducted over two days, as each session lasted approximately three hours. The time required for the study was also a primary reason patients dropped out or declined to participate. As a further limitation, the measures tested in this study primarily provide information on motor output, and altered sensory pathways resulting from the loss of ACL proprioceptive input would remain undetected [123]. Although all included participants had a pre-injury Tegner score above 5 (indicating involvement in competitive or recreational sports), Tegner values were not analysed. In addition, questionnaires assessing psychological readiness (e.g., Anterior Cruciate Ligament-Return to Sport After Injury) were not collected; future research could address this to determine whether the observed corticospinal adaptations correlate with patients’ subjective confidence and fear of re-injury.

One last point worth discussing regards the VA assessment. In the present study, the resting twitch recorded before the MVC was used to calculate the ITT, whereas previous research by Folland has recommended using the potentiated resting twitch recorded after the MVC [124]. This potentiation, which likely affects both the resting twitch recorded after MVC and the active one during MVC, is strictly peripheral and primarily driven by Type II muscle fibres. As already mentioned, ACL reconstruction is associated with selective atrophy of Type II fibres on the operated limb. Since potentiation depends on Type II fibre integrity, normalising the (potentiated) superimposed twitch to the (unpotentiated) pre-MVC resting twitch may lead to a relative overestimation of the operated side’s VA. However, we observed no significant inter-limb differences in VA in our study. Therefore, this potential methodological artefact did not yield a false-positive result (i.e., a spurious asymmetry). The fact that we did not observe significant differences—even with a method that might theoretically inflate the operated side’s VA—suggests that our finding of equal VA is robust and not an artefact. This result is consistent with biomechanical and neurophysiological findings (e.g., increased corticospinal excitability), suggesting that the operated side may require greater neural drive—other than an increased VA—to compensate for peripheral deficits. In addition, Folland’s findings apply to healthy controls. In an asymmetric, pathological cohort, as the one tested here, the magnitude of twitch potentiation relies on multiple additional factors [125] (e.g., altered fatigue resistance, unknown potentiation kinetics in atrophic muscles, more “sluggish” tension transmission [126]) compared to the pre-MVC resting twitch. Therefore, normalising to the pre-MVC resting twitch eliminates this source of confounding variance and, in our opinion, represents the most conservative approach for this specific pathological model.

That being said, the strengths of the present study can be highlighted.

First, this is the only study to provide measures of muscle trophism, knee joint extensor RFD and maximum torque, quadriceps VA, spinal excitability, and intracortical and corticospinal excitability in the thigh muscles from the same sample of patients with ACLR. In addition, this is the first study to perform neurophysiological testing on lower-limb muscles that are not directly involved in knee mechanics. This is relevant because previous research suggests that alterations in activation and trophism can affect the ankle muscles in patients with ACL injuries [64].

Second, in this study, we employed a sequential recruitment approach, and the sample of participants was highly homogeneous with respect to surgical technique, graft type, time since surgery, age, and sex. Furthermore, we controlled for meniscal procedures and other lower-limb injuries and disorders that, on the one hand, may contribute to joint pain and muscle weakness, and, on the other hand, are suspected to induce changes in corticospinal excitability [127].

Third, a method for assessing corticospinal excitability is proposed here, which involves analysing the way MEP amplitude changes with varying stimulation intensity in the middle portion of the recruitment curve. To the authors’ knowledge, this is the first study to employ LMM to analyse lower limb MEPs in ACLR patients. LMM allowed us to deal with the nested structure of the data and account for both between-subject and within-subject variability, considering the random and fixed effects. However, we are aware of two previous studies that have analysed quadriceps MEPs with linear regression in patients with a history of ACL injury [128] or ACLR [42]. Apart from the differences in the statistical analysis, it is also important to highlight that, in the two studies, linear regression lines were fitted on the MEPs in the range of stimulation intensities between 90% and 140% of the active motor threshold [42], or on all the data points between the rMT and the “point of saturation” (likely the beginning of the upper plateau) [128]. Therefore, in both those papers, MEPs from the lower plateau, ascending ramp, and possibly the upper plateau were included in the analysis, without distinguishing between data from linear and non-linear portions of the sigmoid curve. As previously discussed, the MEPs that belong to the lower plateau (i.e., adjacent to the motor threshold) and those from the middle portion of the curve likely describe different properties of corticospinal excitability, with the steepness of the ascending curve representing the gain of the MEP amplitude-stimulation intensity relationship [65]. However, possible drawbacks of our method, which need to be further investigated, are that large numbers of stimulation intensities may need to be explored to build a reliable recruitment curve, and that the whole shape of the sigmoid curve can’t always be identified due to technical limitations (i.e., being the maximum stimulation output delivered by the TMS device an impassable limit).

## Conclusions

This cross-sectional study confirms that quadriceps atrophy persists on the operated side in young male patients even 6–12 months after ACLR. However, this morphological deficit appears to be counterbalanced by specific neurophysiological adaptations. LMM analysis of recruitment curves revealed an increased gain in the corticospinal pathway directed to the operated VM, suggesting a compensatory upregulation of neural drive. Consistent with this enhanced excitability, the operated knee extensors exhibited a preserved—and even increased—capacity for rapid force production (early-phase RFD), although not an increased maximal force output. Collectively, these findings suggest that recovery after ACLR relies on complex central reorganisations to compensate for peripheral impairments. These metrics warrant further investigation in larger cohorts and could pave the way for novel assessment strategies and therapeutic interventions, potentially combining resistance training with neuromodulation techniques to directly target neuroplasticity [129]. 

## Supplementary Information

Supplementary Material 1.

## Data Availability

The raw data supporting the conclusions of this article are available from the authors upon reasonable request.

## Abbreviations

ACL: Anterior cruciate ligament
ACLR: Anterior cruciate ligament reconstruction
BMI: Body mass index
cMAP: compound muscle action potential
CNS: Central nervous system
CSA: Cross-sectional area
EMG: Electromyography
EPSP: Excitatory post-synaptic potential
GABA: Gamma-aminobutyric acid
ICF: Intracortical facilitation
ITT: Interpolated twitch technique
LMM: Linear mixed-effects models
MEP: Motor evoked potentials
MV: Muscle volume
MVC: Maximal voluntary contraction
RFD: Rate of force development
rMT: resting motor threshold
SICI: Short-interval intracortical inhibition
TA: Tibialis anterior
TMS: Transcranial magnetic stimulation
VA: Voluntary activation
VM: Vastus medialis
