# Supplementary material for: Anterior cruciate ligament reconstruction is associated with increased corticospinal excitability and rate of force development
Source: BMC Sports Sci Med Rehabil. 2026 Feb 9;18:123. doi: 10.1186/s13102-026-01577-0 (PMC12983487; doi:10.1186/s13102-026-01577-0)
Supplement: Supplementary file 1 — Supplementary Material 1. [file 13102_2026_1577_MOESM1_ESM.docx]

Supplementary Materials to

Anterior Cruciate Ligament reconstruction is associated with increased corticospinal excitability and rate of force development.

Stefano Scarano^1^, Antonio Caronni^1,2 *^, Alessandra Menon^3,4^, Viviana Rota^1^, Maurizio Amadei^1^, Laura Perucca^1,2^, Elena Brevi^2^, Alessio Maione^5^, Paolo Ferrua^2,5^, Luigi Tesio^1^, and Pietro Simone Randelli^2,5,6^

^1^ IRCCS Istituto Auxologico Italiano, Department of Neurorehabilitation Sciences, Ospedale San Luca, Milan, Italy

^2^ Department of Biomedical Sciences for Health, University of Milan, Milan, Italy

^3^ Department of Biomedical Sciences for Health, Laboratory of Applied Biomechanics, University of Milan, Milan, Italy

^4^ Department of Clinical and Community Sciences, Graduate School of Health Statistics and Biometrics, University of Milan, Milan, Italy

^5^ ASST Gaetano Pini-CTO, U.O.C. 1° Clinica Ortopedica, Milan, Italy

^6^ REsearch Center for Adult and Pediatric Rheumatic Diseases (RECAP-RD), Department of Biomedical Sciences for Health, Università degli Studi di Milano, Via Mangiagalli 31, 20133 Milan, Italy

Correspondence:

Antonio Caronni

[a.caronni@auxologico.it](mailto:a.caronni@auxologico.it)

**Section 1: Description of the surgical procedure**

The following procedure was followed during surgery. The patient was placed in a supine position with a proximal thigh tourniquet and thigh counter-support, and a roller was positioned under the foot to maintain the knee at 90° flexion. A vertical incision was performed medially at the tibial crest to access the semitendinosus and gracilis tendons. After carefully cutting the vincula, the tendons were whip-stitched and harvested using a tendon stripper with the knee flexed at 90°. The tendons were folded into four strands and calibrated. Arthroscopy was performed via superomedial, anterolateral, and anteromedial portals using a pump (60 mmHg). Meniscal tears were addressed if present. The native ACL’s tibial and femoral footprints were identified. A tibial tunnel was drilled using a guide and matched to the graft's diameter. The femoral tunnel was created via a trans-tibial approach, stopping just before the lateral cortex. The graft was looped onto a fixed femoral button and guided through the tibial tunnel into the joint. Once the button was flipped on the femoral cortex, the graft was advanced into the femoral tunnel. The knee was cycled to pre-tension the graft, which was then fixed in the tibial tunnel with a 25 mm interference screw, 1 mm larger than the graft diameter. The procedure concluded with Lachman and pivot-shift tests to confirm the stability of the graft. Two drains were placed into the joint.

**Section 2: Brief description of the recommendations for the post-ACLR rehabilitation protocol**

0–2 Weeks

- Weight-bearing as tolerated with crutches
- Goals: control swelling, achieve full extension, flexion 0–90°.
- Exercises: quad sets, straight-leg raise, heel slides, patellar mobilisation, ankle pumps.

2–6 Weeks: Range of Motion + Activation

- Aim for full extension; progressive flexion to full by 6 weeks.
- Continue gait training; discontinue crutches when safe.
- Strength: closed-chain exercises (mini-squats, step-ups), core, glutes.
- Avoid resisted hamstring curls until ~6 weeks to protect the graft harvest site.

6–12 Weeks: Strength & Neuromuscular Control

- Progressive strengthening: leg press (0–60°), bridges, wall sits.
- Begin light hamstring strengthening (curl variations as tolerated).
- Balance/proprioception training (single-leg stance, wobble board).
- Cardiovascular: cycling, elliptical.

3–6 Months: Advanced Strength + Running

- Advance resistance training and single-leg work.
- Begin a graded running program in line (after 4th month).
- Introduce low-level plyometrics (hops, light jump training).
- Continue agility drills (non-contact direction changes).

6–9+ Months: Return to Sport

- High-level plyometrics, sprinting, cutting drills.
- Sport-specific training with progressive intensity.
- Objective testing before clearance: strength symmetry (>90%), hop tests, good movement mechanics.

**Section 3: Details on the procedure for measuring thigh length, quadriceps muscle thickness, and mid-thigh circumference**

Following Miyatani et al. (1), the positions of the greater trochanter of the femur and the lateral articular cleft between the femoral condyles and the tibia were manually identified and marked with a dermatographic pen. Then, with the participant standing with his arms and legs relaxed, the thigh length, thigh circumference, and muscle thickness were measured.

The thigh length was measured, with a tape measure, as the distance between the greater trochanter of the femur and the lateral articular cleft of the knee, as previously marked on the skin.

The skin was also marked at 50% of thigh length, and thigh circumference was measured at this location with a tape measure.

For measuring the thickness of the knee extensors, an ultrasound transducer scanning head (Esaote MyLab™30, equipped with a 10 MHz linear-array ultrasound transducer LA523) was placed, in correspondence with 50% of the thigh length, on the skin of the anterior surface of the thigh, perpendicular to the underlying muscle and bone tissues. A water-soluble transmission gel was applied to the skin to provide acoustic contact, and care was taken to avoid depressing the dermal surface. The cross-sectional ultrasonic image was saved. In the saved image, the interfaces between subcutaneous adipose tissue and muscle, and between muscle and bone, were identified. The distance from the adipose tissue-muscle interface to the muscle-bone interface was measured and used as a representative of the muscle thickness of the knee extensors.

**Section 4: Details on** **instruments and settings for knee extensors’ strength and voluntary activation testing**

Strength and muscle activation tests were performed using a Cybex Humac Norm© 2014 isokinetic dynamometer (CSMi Computer Sports Medicine, Inc., Stoughton, MA, USA). During testing, the participant sat upright on the dynamometer, with the hip flexed at approximately 90°. The lateral epicondyle of the femur was aligned with the dynamometer's horizontal axis of rotation, and the lower limb was secured to a Johnson anti-shear device. (2) Bilateral restraints were applied to the torso, and a Velcro strap was secured to the distal thigh of the tested limb to limit any unwanted movement.

The patient was instructed to grasp the ad hoc seat handles during testing. Joint moments were displayed on a computer screen, allowing the examiner to visually assess the patient’s performance in real time. The participant was instructed to disregard the screen and focus on perceiving his/her own effort. Isometric tests were conducted with the knee held at 40° flexion (0° = full extension).

In this study, for interpolated twitch technique (ITT) testing, electrical stimulation was delivered via percutaneous rubber electrodes (120 × 220 mm), with the anode positioned on the anterior aspect of the upper thigh and the cathode on the anterior aspect of the lower thigh. Through a constant current high-voltage stimulator (Digitimer© DS7A, Hertfordshire, UK), a doublet of single square-wave stimuli (duration about 50-100 μs, amplitude about 0.3-0.6 A, interstimulus interval 10 ms) was delivered. (3–5) The procedure is painless and is described in full elsewhere. (6)

The amplitude of the electrical stimuli was tailored for each participant. Testing began with the healthy (i.e., non-operated) limb, as it was a priori assumed that this limb would yield the highest torque values in the knee extensors. After a habituation phase of 3 – 4 submaximal knee extension isometric contractions at increasing effort, a maximal voluntary contraction (MVC) was requested. This MVC was used to define the amplitude of the electrical stimuli: the current amplitude was the one providing, at rest, an isometric peak moment of at least 25% of the MVC. (7–9) This current was used to stimulate both limbs throughout all tests.

**Section 5: Details on transcranial magnetic stimulation testing**

During transcranial magnetic stimulation (TMS) testing, the figure-of-eight coil was positioned over the participant’s head, and TMS of the lower limb area of the contralateral motor cortex evoked MEPs.

First, the “hot spot”, i.e., the location on the head that elicited the greatest MEP to the contralateral muscle, was identified. The location of the hot spot corresponded to the position and orientation of the coil providing the largest MEP responses, as recorded from muscles contralateral to the examined hemisphere. (10) The position of the coil corresponding to the hot spot was marked on the participant’s skin with a dermatographic pen. All MEPs were delivered with the coil manually positioned over the hotspot, and skin markings ensured consistent coil placement throughout the trials.

Following the identification of the hot spot, the rMT was measured. For each of the investigated muscles, the rMT was determined, with the limb at rest, as the lowest intensity of magnetic stimulation that was required to evoke a MEP with a peak-to-peak amplitude ≥ 50 µV in five out of ten consecutive trials. (10)

Subsequently, TMS stimuli were delivered to construct the recruitment curves. Initially, eight stimulation intensities were tested per muscle, randomly selected between 20% and 100% of the maximum stimulator output (MSO) in 5% increments. For each muscle, the operators controlled whether the upper plateau of the recruitment curve had been identified. To do so, they checked whether MEPs evoked at the highest MSO tested had a greater amplitude than those preceding them. In case the upper plateau had not been clearly explored, up to five additional stimulation intensities were tested.

During single-pulse TMS for recruitment curves assessment, the degree of muscle contraction was standardised as follows: first, the participant was asked to perform a maximal voluntary contraction against resistance, and the corresponding EMG activity was online recorded; then, the participant was requested to perform submaximal voluntary muscle contractions, achieving an EMG amplitude corresponding to 20% of the one recorded during maximal contraction. Acoustic EMG feedback was provided throughout the whole testing to help the participant achieve and maintain the desired level of effort.

During testing, frequent pauses were included (approximately every 40 seconds, lasting one minute) to avoid fatigue. In addition, for all TMS testing (both single-pulse and paired-pulse TMS), stimulations were delivered at a rate of < 0.2 Hz to prevent the conditioning of cortical excitability. (11)

**Section 6: Absolute and normalised peak torque values**

| **ID** | **Absolute peak torque - Operated side**  **(Nm)** | **Absolute peak torque - Healthy side**  **(Nm)** | **Normalised peak torque (CSA) - Operated side**  **(Nm cm^-2^)** | **Normalised peak torque (CSA) - Healthy side**  **(Nm cm^-2^)** | **Normalised peak torque (Weight) - Operated side**  **(Nm kg^-1^)** | **Normalised peak torque (Weight) - Healthy side**  **(Nm kg^-1^)** |
| --- | --- | --- | --- | --- | --- | --- |
| 1 | 123.4 | 119.3 | 3.11 | 2.77 | 2.08 | 2.01 |
| 2 | 194.6 | 174.5 | 3.45 | 3.11 | 2.32 | 2.08 |
| 3 | 110.1 | 115.8 | 2.34 | 2.43 | 1.85 | 1.95 |
| 4 | 143.2 | 179.8 | 3.30 | 3.52 | 1.84 | 2.31 |
| 5 | 117.7 | 131.7 | 2.52 | 2.47 | 1.60 | 1.79 |
| 6 | 118.6 | 118.3 | 2.87 | 2.75 | 1.79 | 1.79 |
| 7 | 142.3 | 142.8 | 3.12 | 2.76 | 1.81 | 1.82 |
| 8 | 152.2 | 173.6 | 3.81 | 3.29 | 1.85 | 2.10 |
| 9 | 116.0 | 140.5 | 2.29 | 2.71 | 1.55 | 1.87 |
| 10 | 265.1 | 228.6 | 4.55 | 3.92 | 3.08 | 2.65 |

**Section 7: Effect sizes with 95% confidence intervals**

| **Measure** | **Effect size (r)** | **95% confidence interval** |
| --- | --- | --- |
| Volume of the knee extensors | 0.807 | [0.57, 0.89] |
| Thigh circumference | 0.873 | [0.81, 0.90] |
| Maximum normalised mean torque | 0.435 | [0.05, 0.89] |
| Voluntary activation | 0.145 | [0.02, 0.74] |
| Tibialis anterior resting motor threshold | 0.066 | [0.02, 0.76] |
| Vastus medialis resting motor threshold | 0.098 | [0.02, 0.72] |
| Tibialis anterior maximum mean MEP | 0.081 | [0.02, 0.70] |
| Vastus medialis maximum mean MEP | 0.467 | [0.02, 0.83] |
| Hmax/Mmax ratio | 0.048 | [0.02, 0.70] |

**Section 8: Estimates and 95% confidence intervals for the slopes of the LMM models**

RFD analysis: the estimated [95% CI] slopes of the LMM models describing the relationship between torque increment and time from onset were 513.96 [421.82, 606.10] and 665.19 [573.06, 757.33] for the healthy and operated limbs, respectively.

Recruitment curves analysis: the estimated [95% CI] slopes of the LMM models describing the relationship between MEP amplitude and stimulation intensity were as follows: 1.27 [1.09, 1.45] and 1.47 [1.22, 1.71] for the healthy and operated limb’s TA, respectively; 0.53 [0.39, 0.67] and 1.47 [1.25, 1.69] for the healthy and operated limb’s VM, respectively.

SICI analysis: the estimated [95% CI] slopes of the LMM models describing the relationship between MEP amplitude and the condition (i.e., conditioned or unconditioned stimulations) were as follows: 3.73 [2.77, 4.69] and 2.38 [1.42, 3.35] for the healthy and operated limb’s TA, respectively; 0.49 [0.30, 0.68] and 0.55 [0.36, 0.74] for the healthy and operated limb’s VM, respectively.

**Section 9: missing data in the analysis of MEP recruitment curves and SICI**

For four recruitment curves, concerning a) the healthy side TA muscle of one participant, b) the operated side TA and c) VM muscles of a second participant, and d) the operated side VM muscle of a third participant, only four MEP data points (i.e., one stimulation intensity, only) were included between the 18% and 82% boundaries. This was due to the randomisation of stimulation intensities applied during testing (see Section 5 of the Supplementary Materials), as fewer intensities were tested in the central portion of the curve for these muscles. These data were included in the model, along with the others.

In the SICI analysis, for one participant, due to technical issues, only eight unconditioned stimulations were recorded for the operated side TA muscle, while four conditioned and five unconditioned stimulations were recorded for the operated side VM. The available data were entered into the models.

**References**

1. Miyatani M, Kanehisa H, Ito M, Kawakami Y, Eukunaga T. The accuracy of volume estimates using ultrasound muscle thickness measurements in different muscle groups. Eur J Appl Physiol. 2004 Mar;91(2–3):264–72.

2. Li CK, Chan KM, Hsu SYC, Chien P, Wong MWN, Yuan Y. The Johnson antishear device and standard shin pad in the isokinetic assessment of the knee. Br J Sports Med. 1993;27(1):49–52.

3. Suter E, Herzog W. Effect of number of stimuli and timing of twitch application on variability in interpolated twitch torque. J Appl Physiol (1985) [Internet]. 2001 Mar;90(3):1036–40. Available from: http://www.ncbi.nlm.nih.gov/pubmed/11181617

4. Bampouras TM, Reeves ND, Baltzopoulos V, Maganaris CN. Muscle activation assessment: Effects of method, stimulus number, and joint angle. Muscle Nerve. 2006 Dec;34(6):740–6.

5. Folland JP, Williams AG. Methodological issues with the interpolated twitch technique. Journal of Electromyography and Kinesiology [Internet]. 2007 Jun;17(3):317–27. Available from: https://linkinghub.elsevier.com/retrieve/pii/S1050641106000691

6. Catino L, Malloggi C, Scarano S, Cerina V, Rota V, Tesio L. Quadriceps activation during maximal isometric and isokinetic contractions: The minimal real difference and its implications. Isokinet Exerc Sci. 2021 Jan 1;29(3):277–89.

7. Pap G, Machner A, Awiszus F. Strength and voluntary activation of the quadriceps femoris muscle at different severities of osteoarthritic knee joint damage. Journal of Orthopaedic Research. 2004;22(1):96–103.

8. Bülow PM, Nørregaard J, Mehlsen J, Danneskiold-Samsøe B. The twitch interpolation technique for study of fatigue of human quadriceps muscle. J Neurosci Methods [Internet]. 1995 Nov;62(1–2):103–9. Available from: http://www.ncbi.nlm.nih.gov/pubmed/8750091

9. Nørregaard J, Lykkegaard JJ, Bülow PM, Danneskiold-Samsøe B. The twitch interpolation technique for the estimation of true quadriceps muscle strength. Clinical Physiology. 1997;17(5):523–32.

10. Rossini PM, Burke D, Chen R, Cohen LG, Daskalakis Z, Di Iorio R, et al. Non-invasive electrical and magnetic stimulation of the brain, spinal cord, roots and peripheral nerves: Basic principles and procedures for routine clinical and research application: An updated report from an I.F.C.N. Committee. Vol. 126, Clinical Neurophysiology. Elsevier Ireland Ltd; 2015. p. 1071–107.

11. Zarzycki R, Morton SM, Charalambous CC, Pietrosimone B, Williams GN, Snyder-Mackler L. Athletes after anterior cruciate ligament reconstruction demonstrate asymmetric intracortical facilitation early after surgery. Journal of Orthopaedic Research. 2021 Jan 1;39(1):147–53.
